# Epidermal growth factor receptor gene mutations in patients with lung adenocarcinoma differ by frequency and type between Uighur and Han ethnic groups in Xinjiang Autonomous Region

**DOI:** 10.1186/s12863-015-0181-4

**Published:** 2015-02-28

**Authors:** Xiaoqin Li, Xiuli Wang, Hongge Zhu, Chunling Liu, Xin Zhou, Bing Zhao, Huijie Duan, Jia Yang, Guomin Gu, Yiyi Zhan, Jing Yuan, Kahaer Abuduwaili, Su Qionglu

**Affiliations:** Department of Pulmonary medicine, The Third Affiliated Hospital of Xinjiang Medical University, Wulumuqi, 830011 China

**Keywords:** Lung adenocarcinoma, EGFR gene mutation, Uighur nationals

## Abstract

**Background:**

This study was designed to investigate epidermal growth factor receptor (EGFR) mutation types affecting lung cancer treatment in patients in Xinjiang, China. We detected and analyzed differences in the EGFR mutation points of Uighur and Han patients with lung adenocarcinoma. We examined 181 specimens of lung adenocarcinoma tissue embedded with paraffin (76 Uighur and 105 Han patients) for mutations in the EGFR gene in exon 18-21 by the amplification refractory mutation system (ARMS) method. We used the chi-square statistical method to analyze the relationship between mutations and patients’ clinical parameters.

**Results:**

EGFR somatic mutations were detected in 59 of 181 cases (32.6%). The mutation rate was higher in Han patients (45.7%) than in Uighur patients (15.8%) (*P* < 0.001). The main mutation types were the exon 19 deletion and the L858R point mutation in exon 21. In Han patients we found 21 (44.7%) cases of exon 19 deletion, 24 (51.1%) cases of L858R in exon 21, 1 case (2.1%) with mutations in both exon 19 and exon 21, and 1 case (2.1%) with T790 mutation in exon 20. In Uighur patients we found 8 (66.7%) cases of exon 19 deletion and 4 (33.3%) cases of L858R in exon 21.

**Conclusions:**

In comparing these groups, the exon 19 deletion was more common than L858R in exon 21 in Uighur patients. In Han patients, EGFR-sensitive mutations occurred in female, never-smoking patients with well-differentiated tumors; but for Uighur patients only smoking history showed an obvious correlation.

## Background

Lung cancer has become the leading cause of cancer death worldwide, and the incidence of lung cancer is still on the rise in most countries. Five-year overall survival is only 10% in China. In 2010, an estimated population of 222,500 patients was newly diagnosed with lung cancer and bronchiolar carcinoma (116,750 males and 105,770 females), with 157,300 deaths (86,200 males and 71,100 females) [1].

Recently, molecular targeting therapies against epidermal growth factor receptor (EGFR) have gained increasing recognition in the treatment of lung cancer. Treatment with EGFR tyrosine kinase inhibitor (TKI), e.g. gefitinib and/or erlotinib, in advanced lung cancer has proven itself effective. Multiple clinical studies [2-4] have suggested that female Asian patients with adenocarcinoma who had never smoked had superior efficacy with gefitinib and erlotinib, particularly those with bronchioloalveolar carcinoma (BAC). Study results demonstrated that the EGFR tyrosine kinase inhibitor gefitinib (Brand name Iressa®) achieved a response rate of more than 80% in mutant tumors, but was basically ineffective in wild-type tumors without mutations [5]. These results were confirmed post-publication by scientists in other countries. Therefore, guiding EGFR-TKI therapy with EGFR gene status as predictive molecular marker is an important and practical strategy.

Research shows that EGFR TKI therapy efficacy and EGFR gene mutation status are significantly related to the patients’ ethnic or regional origin and can vary vastly [6]. For example, the exon 21 mutation predominated in Taiwan, and the exon 19 mutation predominated in Guangdong region, but no significant difference was noted between mutations in these two exons in Beijing [7-9]. Most of the studies in this field have focused on populations in Europe and East Asia, but rarely on those in the Middle East and Middle Asia. Xinjiang is located at the junction of the Eurasian continent, and recently the incidence of lung cancer among the local Uighur population has increased year by year. Because EGFR gene mutation status in Uighur patients with lung adenocarcinoma is not known, we designed this study to provide genetic evidence for effective treatment with EGFR-TKI therapy.

## Results and Discussion

### Patient characteristics

Among 181 lung adenocarcinoma patients, the EGFR mutation rate was 32.6%. In the group of 76 Uighur patients, the EGFR mutation rate was 15.8%; and in the group of 105 Han patients, the mutation rate was 44.8%. For all types of EGFR mutations, the rates were significantly lower in the Uighur patients than in the Han patients (P < 0.001) (Table 1). The two main types of mutation were an exon 19 deletion mutation and L858R (base substitution mutation in codon 858). The percentage of Uighur patients with the exon 19 mutation (66.7%) is higher than that with the exon 21 mutation (33.3%). By contrast, the percentage of Han patients with the exon 19 mutation (44.7%) is slightly lower than that with the exon 21 mutation (51.1%). In the Han group, one patient had both exon 19 and exon 21 mutations (2.1%), and one patient had a T790 mutation in exon 20 (2.1%).

**Table 1:** **EGFR genetic mutation rates in lung adenocarcinoma patients of Uighur and Han ethnic groups**

**Ethnic group**	**No. of patients**		**EGFR gene**	
		**No. of patients with no mutation(%)**	**No. of patients with mutation(%)**	**P value**
Uighur ethnic group	76	64(84.2)	12(15.8)	P < 0.001
Han ethnic group	105	58(55.2)	47(44.8)	

### Relationship between EGFR genetic mutation and clinicopathological features in adenocarcinoma patients of Han ethnic group

In the Han group, female patients had a significantly higher EGFR genetic mutation rate as compared to male patients, with statistically significant difference between them (P < 0.05). Patients with no smoking history had a significantly higher EGFR genetic mutation rate as compared to smoking patients, with statistically significant difference between them (P < 0.05). Patients with well-differentiated tumors had significantly higher EGFR genetic mutation rates as compared to patients with poorly differentiated tumors, with statistically significant difference between them (P < 0.05). No statistically significant correlations were found between EGFR genetic mutation rate and either the patients’ age group or staging (P > 0.05) (Table 2) [2].

**Table 2:** **Relationship between EGFR genetic mutation and clinicopathological features in patients of Han ethnic group**

**Characteristics**		**EGFR**			
		**Mutations**	**Wild**	***χ*** ^**2**^	**P**
Sex	Male	19	37	5.696	0.017
	Female	28	21		
Age	≤65	24	47	0.282	0.595
	>65	18	16		
Smoking	Yes	18	34	4.289	0.034
	No*	29	24		
pTNM stage	≤IIIa	12	13	0.354	0.552
	>IIIa	33	47		
Tumor differentiation	Well-differentiated	41	35	5.861	0.015
	Poorly differentiated	8	21		

*No smoking is defined as: previous total amount of smoking fewer than 100 cigarettes.

### Relationship between EGFR genetic mutation and clinicopathological features in adenocarcinoma patients of Uighur ethnic group

Uighur patients with no smoking history had a significantly higher EGFR genetic mutation rate as compared to smoking patients, with statistically significant difference between them (P < 0.05). When we analyzed the relationship between EGFR genetic mutations and clinicopathological features in adenocarcinoma patients, we found no correlations in terms of sex, age groups, staging and tumor differentiation (P > 0.05) (Table 3) [3].

**Table 3:** **Relationship between EGFR genetic mutation and clinicopathological features in patients of Uighur ethnic group**

**Characteristics**		**EGFR**			
		**Mutations**	**Wild**	***χ*** ^**2**^	**P**
Sex	Male	7	42	0.235	0.628
	Female	5	22		
Age	≤65	8	41	0.09	0.764
	>65	4	25		
Smoking	Yes	3	38	4.806	0.028
	No*	9	26		
pTNM stage	≤IIIa	3	15	0.014	0.907
	>IIIa	9	49		
Tumor differentiation	Well-differentiated	10	36	3.102	0.078
	Poorly differentiated	2	28		

*No smoking is defined as: previous total amount of smoking fewer than 100 cigarettes.

EGFR gene mutation can lead to amplification and overexpression of EGFR protein as well as to other carcinogenic mechanisms of EGFR tyrosine kinase activity disorder. Inappropriate activation of EGFR tyrosine kinase can promote tumor angiogenesis, tumor cell proliferation, adhesion, invasion and metastasis. In theory, specific blocking of the receptors can inhibit tumor formation and angiogenesis, and this theory has been confirmed in clinical studies [5,6]. However, in a small sample survey conducted in our hospital, we observed that of 15 Uighur patients with chemotherapy failure in treatment of non-small cell lung cancer who received molecular targeted drugs, only 2 of the patients showed stable disease, while 13 patients showed progressive disease. This result sharply contrasts with the satisfactory effect of targeted drugs in the clinical research of lung cancer elsewhere in China, and we hypothesized that the difference may indicate a geographical and ethnic variation in the incidence and type of mutation.

Research shows that EGFR mutations in adenocarcinoma in different ethnic groups varied substantially. Schmid et al. [10] and Kris et al. [11] found the rate of Caucasian EGFR mutations in lung cancer is about 7% -17%. In a study of 202 lung cancer patients, Li et al. [12] found that the rate of Han lung adenocarcinoma EGFR mutation was 75.3%. Han [13] and Hsu et al. [14] further showed that the rate of EGFR mutations in lung adenocarcinoma patients of East Asian ethnicities is about 30% -62%. In this study, the Uighur lung adenocarcinoma EGFR mutation rate was slightly higher than that reported in the literature on Caucasian populations ( [2,5,15], 3-13%), and was significantly lower than that of the local Han cases (15.8% vs 44.8%) [12,16,17]. The rates of EGFR mutations in lung adenocarcinoma showed a gradually increasing trend from Europe to the East Asian continent through the geographical distribution of these ethnic groups: the Han ethnic group is from the East Asian continent, Caucasians are from the European continent, and Uighurs live between Europe and the East Asian continent. Further study has confirmed that different racial genetics decide and influence the prognosis and response to treatment. Uighur lung cancer patients with targeted therapy are associated with lower mutation rates.

Research shows that tumor response to erlotinib is greater in patients with exon 19 mutation compared with patients with exon 21 mutation [18]. In our study, the Uighur group’s mutation rate in exon 19 was higher than that in exon 21 (66.7% vs 33.3%), which is similar to Caucasian population [18]. The Han patients, however, had a slightly lower percentage of exon 19 mutations than exon 21 mutations (44.7% vs. 51.1%). The insertion mutation in exon 20 was not found in the Uighur patients. In the Uighur population the overall effect of EGFR TKI therapy may be better than in the Han population.

Previous reports indicate that EGFR mutation is closely related to age, sex, smoking status, and tumor differentiation. EGFR mutations are common in older, female, non-smoking patients with well-differentiated tumors (especially bronchioloalveolar carcinoma) [12,17,18]. Our study’s findings are in accordance with these reports in the Han group: EGFR mutation associated with sex, smoking history, and the degree of tumor differentiation. In the Uighur group, EGFR mutations were correlated with smoking history. The relationship between gene mutation rate and sex or degree of tumor differentiation is not clear, perhaps because the Uighur patients in our study were mostly male and were a small group.

## Conclusion

The EGFR mutation rate in Uighur patients with lung adenocarcinoma is relatively lower than in Han patients, perhaps because of differences in race and region. Uighur patients’ mutation ratio is close to that reported in Caucasian patients, which may be the main reason for poor treatment effectiveness in Uighur patients. Genetic testing for mutations in Uighur patients before treatment could prevent waste of therapeutic resources and could specify targeted drug therapy. In cases of limited resources, where no EGFR gene detection is available and the patient shows intolerance to chemotherapy, smoking history is a reference index. Because of the small number of patients in this study, the relationship between other clinical factors and EGFR mutation remains to be verified by larger-scale studies.

## Methods

### Study participants and tissue samples

The tissue samples examined in this study were collected by biopsy from November 2011 to January 2014 in Xinjiang Tumor Hospital, and all were diagnosed by pathology. Specimens indicating adenocarcinoma of the lung were paraffin-embedded. The study participants were 76 Uighur patients (49 males and 27 females, ages 46- to 78-years-old, with median age 57 years) and 105 Han patients (56 males and 49 females, ages 25- to 85-years-old, with median age 53 years). AJCC TNM staging criteria were used to rate the disease as either stage ≤ IIIa or > IIIa. We used H&E staining to divide the specimens into two groups: well-differentiated tumors and poorly differentiated tumors. The study was reviewed and approved by the Institutional Ethics Committee at Xinjiang Tumor Hospital. All patients signed informed consent forms to participate in this study and to give permission for the use of their tissue.

### Sample collection and DNA extraction

We collected samples and extracted DNA from 5- to 8-mm slices of paraffin section, stored at room temperature. The samples were determined by pathologic diagnosis to contain tumor tissue and were preserved for no more than 2 years. We used DNA extraction kit (QIAGEN, Germany), and we dissolved the extracted DNA in Tris-HCl (10 mmol/L, pH 8.0). After sample quality was checked by UV spectrophotometer (Amoy Diagnostics Co. Ltd, China), we added Tris-HCl solution (10 mmol/L, pH 8.0) to adjust DNA concentration to 10 ng/μL and 2 ng/μL standby.

### ARMS

Real-time PCR amplified by the amplification refractory mutation system (ARMS) method was used to detect mutations in the EGFR gene exons 18-21. All samples were amplified by Strata Gene MX3000P real-time PCR instrument (Amoy Diagnostics Co. Ltd, China) according to kit instructions, with each test designed with positive and negative control groups. If the Ct value = 0, or Ct value > 30, the experiment will result in wild type. The reaction conditions were as follows: 1 cycle of fluorescence PCR pre-degeneration in 95°C for 5 min; 15 cycles degeneration in 95°C for 25 s, 64°C for 20 s, 72°C for 20 s; and 31 cycles 93°C degeneration for 25 s, 60°C for 35 s, 72°C for 20 s.

### Statistical analysis

Data was analyzed using statistical software SPSS 18. Chi-square test was used to compare differences in mutations between the two groups as well as the relationship between patients’ clinical features and EGFR mutations. Probability value was obtained from two-sided tests, with statistical significance defined as P < 0.05.

## Abbreviations

ARMS: Amplification refractory mutation system
BAC: Bronchioloalveolar carcinoma
EGFR: Epidermal growth factor receptor
TKI: Tyrosine kinase inhibitor
